# Ineffective humoral anti-tick IgY-response in birds: reaction against pathogen constituents?

**DOI:** 10.12688/openreseurope.13204.2

**Published:** 2021-09-23

**Authors:** Dieter Heylen, Beatrice Bisaglia, Gerardo Fracasso, Els Prinsen, Wendt Müller, Erik Matthysen

**Affiliations:** 1 Eco-Epidemiology Group, Institute of Tropical Medicine, Antwerp, Belgium; 2 Interuniversity Institute for Biostatistics and Statistical Bioinformatics, Hasselt University, Diepenbeek, Belgium; 3 Department of Biosciences, University of Milan, Milan, Italy; 4 Biology Department, University of Antwerp, Wilrijk, Belgium

**Keywords:** Tick, Ixodes ricinus, bird, Borrelia burgdorferi s.l., IgY, antibody, constituent, immunity

## Abstract

**Background:** Variation in parasite burdens among hosts is typically related to differences in adaptive immunity. Comprehension of underlying mechanisms is hence necessary to gain better insights into endemic transmission cycles. Here we investigate whether wild songbirds that have never been exposed to ticks develop adaptive humoral immunity against endemic *Ixodes ricinus* ticks.

**Methods**: Blue tits were exposed three times in succession to wild *Ixodes ricinus* ticks. For each infestation, serum samples were obtained. An enzyme-linked immunosorbent assay was developed, using tick salivary antigens, in order to quantify the bird’s IgY response against ticks. In addition, at every sampling occasion the birds’ body weight (corrected for body size) and haematocrit level was determined.

**Results:** Individual IgY levels against the ticks’ salivary proteins increased over three consecutive tick infestations, and large among-individual variation was observed. The responses were specifically directed against *I. ricinus*; cross-reactivity against the congeneric tree-hole tick *Ixodes arboricola* was negligibly low. IgY responses did not impinge on tick feeding success (engorgement weight and attachment success). Yet, those birds with the highest immune responses were more capable to reduce the acute harm (blood depletions) by compensating erythrocyte loss. Furthermore, at the end of the experiment, these birds had gained more body weight than birds with lower IgY levels.

**Conclusions: **Latter observations can be considered as an effect of host quality and/or tolerance mechanisms. Birds anticipate the (future) costs of the activation of the immune system by ticks and/or ongoing tick-borne pathogen infections. Furthermore, although unsuccessful against tick feeding, the IgY responses may indirectly protect birds against tick-borne disease by acting against salivary protein secretions on which pathogens rely for transmission.

## Plain language summary

Songbirds are central elements in the ecological networks of ticks, but are heavily overlooked when it comes to elementary biological mechanisms like immune responses against ticks and tick-borne diseases. No studies so far have related individual variation in wild songbird's adaptive immune responses to components of ectoparasite fitness. Although the immune response was specifically targeted against the tick *Ixodes ricinus*, tick feeding success was not reduced and thus birds clearly did not acquire immunological resistance against the ticks after being exposed for a long time. Interestingly, birds with the highest immune responses were more capable to reduce the acute harm and had gained more body weight than birds with lower IgY levels. Latter observations can be considered as an effect of host quality and/or tolerance mechanisms: birds anticipate the (future) costs of the activation of the immune system by ticks and/or ongoing tick-borne pathogen infections. Although unsuccessful against tick feeding, the immune responses may indirectly protect birds against tick-borne disease by acting against salivary protein secretions on which pathogens rely for transmission. Our study can be considered as a primer for future work exploring tick epitopes that can be targeted by bird immune components.

## Introduction

All parasites show a certain degree of host specialization, partly defining the variation in burdens among host species, but also within a single host species, large variation among individuals in levels of parasitism has been proven to be the rule rather than the exception. This individual variation is - at least for parasites that live for longer periods of time in the off-host environment - determined by the following factors: (1) encounter rates, (2) mechanistic specializations in parasites for host finding and resource exploitation, and (3) host behavioural resistance, immunological resistance and susceptibility ( Hudson *et al.*, 2002; Poulin, 2007; Schmid-Hempel, 2011). The latter two especially show extensive individual variation and are known to have a genetic basis, and to be heritable ( Mazé-Guilmo *et al.*, 2014 and references herein). They are therefore considered to profoundly influence the co-evolutionary dynamics between parasite and host, and hence the (genetic) diversity found in natural host and parasite populations. Yet empirical evidence on how and to what extent host resistance - both behavioural and immunological – actually drives the observed variation in infestation levels in the wild, and thus the (co-)evolutionary processes, remains poorly studied in the majority of macro-parasite-wildlife systems.

This also applies to macro-parasites feeding on birds, such as ticks and mosquitos; the by far most important ectoparasites to human health vectoring micro-organisms that cause disease (e.g. Lyme disease, West-Nile virus) ( Hubálek, 2004). In addition to grooming and preening ( Clayton *et al.*, 2010), birds can reduce infestations by avoidance of ectoparasite-rich habitats ( Christe *et al.*, 1994; Moore, 2002). But those first line behavioural defences are far from effective, particularly during the breeding season when birds face high time and energy demands while rearing their offspring, and must exploit parasite-rich habitats ( Heylen *et al.*, 2013; Richner *et al.*, 1993).

However, the second line of defence, the host’s immunological reaction against natural ectoparasites, has received very little attention in birds, especially with regard to ticks ( Davison *et al.*, 2008; De la Fuente *et al.*, 2015; Heylen *et al.*, 2010). To investigate such immune responses, lab experiments are required in which host individuals are repeatedly exposed in order to allow immunity to develop. Intriguingly, in a previous experimental study we show that naïve songbirds did not acquire resistance against *Ixodes ricinus* (L., 1758) during the first months after fledging: tick engorgement weights and cellular immune responses remained unchanged after repeated exposures ( Heylen *et al.*, 2010; Heylen *et al.*, 2015). Despite this apparent lack of immunological resistance, a substantial amount of among-bird variation was observed in the tick’s feeding success and virulence, which still could be shaped by humoral immune responses.

This study focuses on the avian adaptive humoral immune response against ixodid ticks as a potential driver of the among-bird variation in feeding success. For this, we follow-up ticks that have been placed on the birds’ skin, giving them the maximum opportunity to feed. As the development of an effective immunological response needs time, we exposed birds three times in succession over a time period of 25 days. Throughout the experiment, the birds’ physiological health and IgY-antibody response were monitored (see ‘Methods’ section). For the main part of the study we used wild blue tits ( *Cyanistes caeruleus*, L. 1758) and their endemic ticks in order to reproduce the natural songbird-tick interaction. Birds fledged in tick-free aviaries, ensuring they were tick-naïve before entering the experiments and got habituated to humans to reduce stress responses. In this study we put forward three questions: (1) do birds develop an IgY-antibody response specifically against *Ixodes ricinus* salivary antigens, and how strong is the inter- and intra-individual variation in this response? (2) do individual IgY-levels negatively correlate with tick feeding success (i.e. anti-tick resistance) and (3) how do they correlate with changes in bird physiology due to tick feeding?

## Methods

### Ethical statement

All procedures, including the tick infestation (for more details see below), were carried out in accordance with national environmental legislation and regulations, and were approved by the Ethical Committee for Animal Experiments of the University of Antwerp (Licenses N° 2009-32 and 2016-88). Wild birds were captured under licences N° S8/VERG/07-U5R26 and ANB/BL/FF-V17-00029 of the Agency for Nature and Forests, Flemish Government, Belgium. Bird individuals were kept in optimal conditions at the University of Antwerp, with food and water *ad libitum* in large cages (surface floor 40 cm x 80 cm; height: 40 cm) and had the opportunity to take a bath in fresh water. Birds were monitored daily. Wild birds were released after a minimum time period in captivity. Manipulations of a bird (infestation, blood sampling, weighing, measurement of tarsus length) occurred in a separate section of the lab room, outside the view of the other birds. As manipulations (see below) cause mild distress or harm, the use of analgesics was not necessary.

### Bird serum samples

**Figure 1.**
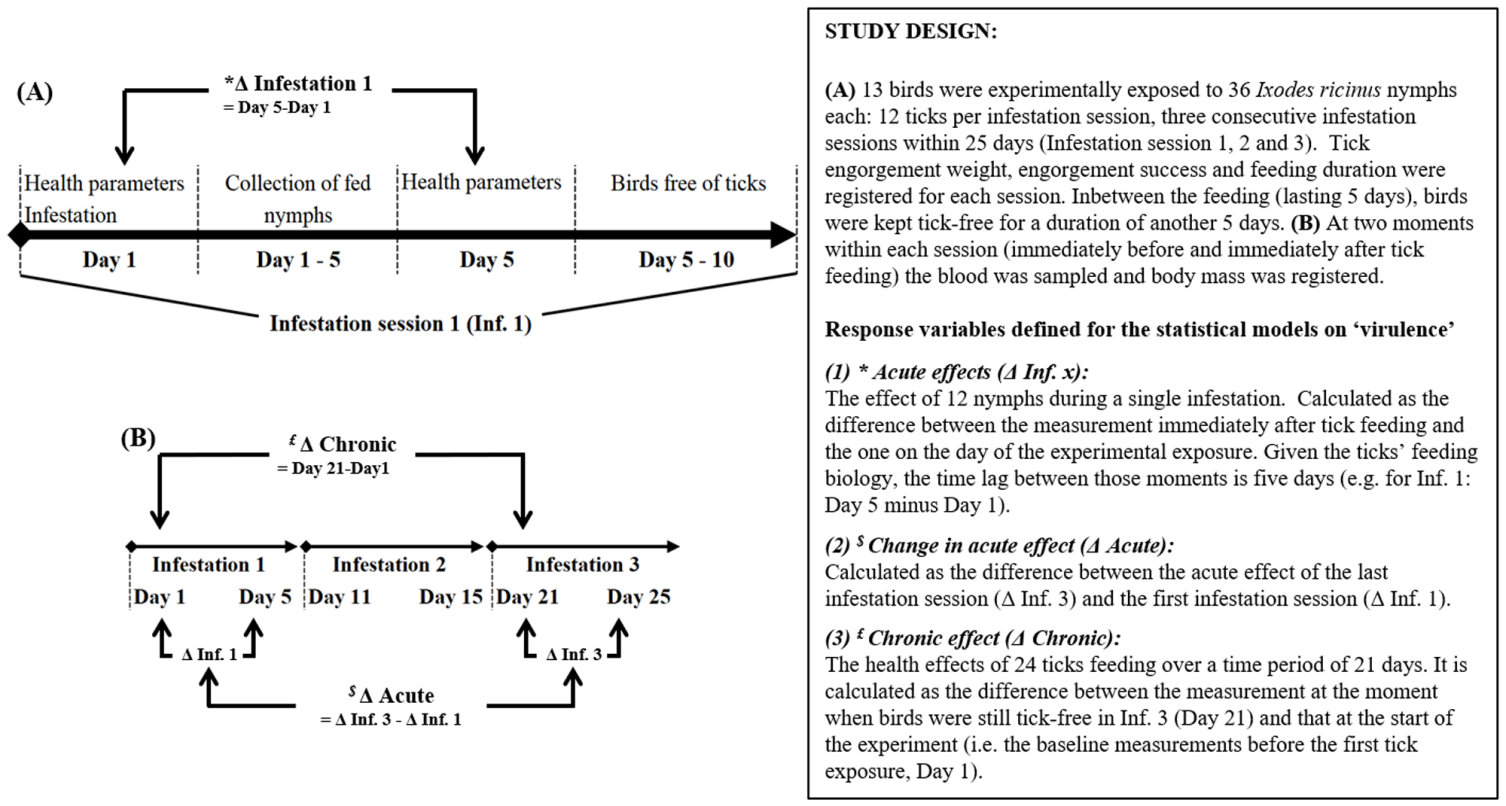
Schematic overview of the study design.

Sera from tick-exposed birds were obtained from 16 blue tits that were exposed three times in succession with 12 nymphs over a time span of 30 days in the summer of 2008 (see Figure 1 for schematic overview of study design). All of them made part of a previous ethically approved experiment and were in good condition ( Heylen *et al.*, 2010). Birds were kept in tick-free aviaries since hatching, thus naïve to ticks at the start of the experimental exposure. A blood sample (maximum 65 μL) was taken from the ulnar vein collected into 75 μL heparinized capillary tubes and subsequently centrifuged for 10 min at 14,000 g, after which the serum was separated from the blood clot and stored in Eppendorf tubes at -80°C until further analysis. To this end, the vein was superficially punctured with a needle (27G). Due to the small body size of the songbirds under study, the sampled serum volumes were kept to a minimal. As the minimum requirement for biochemical analyses was approximately 30 µL serum/bird per sampling occasion, only a limited number of birds (16) of the original experiment (31, see Heylen *et al.*, 2010) could enter the longitudinal analyses (i.e. enough volume in three consecutive infestation sessions). 

### Monitoring of repeatedly exposed blue tits for sero-conversion and physiological changes

When blue tits were nine weeks old, individuals were infested with *I. ricinus* nymphs three times in succession (Infestation 1–3) (see Figure 1 for schematic overview of study design). Each infestation lasted 4–5 days, and the birds were kept free of ticks for a duration of 5–6 days between the consecutive infestations. We infested birds with tick loads corresponding to the maximum level found under natural conditions in our study population ( Heylen *et al.*, 2013). To this end, 12 randomly sampled *I. ricinus* nymphs were put underneath the feathers on the head of each bird in each infestation session using moistened tweezers ( Heylen & Matthysen, 2008). To this end, for each bird, Eppendorf tubes containing a nymph each, were randomly picked out of a box containing the remaining tubes with ticks. Birds were then kept for 2 h in an air-permeable cotton bag (size: 20 cm × 15 cm) inside a darkened cage, which kept them inactive. After tick exposure, birds were placed in individual cages with a wire-mesh floor (40 cm × 80 cm). Below the wire-mesh was a plastic tray containing damp filter paper and edges were streaked with vaseline to prevent nymphs from escaping. The engorged nymphs that dropped through the mesh cage were collected each day with minimal disturbance to the host ( Heylen *et al.*, 2010).

We estimated the effects of the IgY-response (ELISA described below) on tick measures as the change between exposure 1 and exposure 3. We measured the effect of IgY-response on the health measures as the change in health status (described below) between the moments immediately before tick exposure 1 and immediately before tick exposure 3 (i.e. 5–6 days after Inf. 2). Furthermore, we studied the change in the acute responses (i.e. the change in health status immediately before and just after tick exposure; Figure 1A) between infestation 1 and infestation 3 ( Figure 1B) in response to the cumulative IgY response (i.e. the summed OD’s; see further).

We measured two parameters reflecting the hosts’ health status immediately before and after the infestation ( Figure 1A). (1) Haematocrit (Hct) level: anaemia, as indicated by low Hct (the volume percentage of erythrocytes in the blood), results in a reduced oxygen-carrying capacity of the blood and restricts oxygen-demanding processes ( Dein, 1986). Reticulocytes (i.e. immature erythrocytes) are stored in bird bone marrow, and can be instantly released in the blood stream ( Martinho, 2012) upon sudden erythrocyte reduction (e.g. injury). Heparinized capillary tubes containing blood samples were centrifuged for 10 min at 14,000 g, and the ratio of packed red blood cells to the total volume was measured with a digital calliper to the nearest 0.01 mm under optimal light conditions. (2) Body condition (mass/tarsus ratio): body mass was measured to the nearest 0.1 g using a digital balance. To this end, the bird was immobilised by gently placing it in a tube (6 cm length, diameter 2.5 cm).

We subsequently calculated the ratio between body mass and a skeletal measurement (tarsus length, measured with a digital calliper to the nearest 0.01 mm) as a measure of body condition ( Yom-Tov, 2001). Metabolic processes, e.g. for the compensation of parasite harm and mounting immune responses are known to be energy demanding and may lead to a reduced body condition when anabolic processes are hindered (e.g. restricted food conditions, constrained metabolic pathways, etc.) ( Martin *et al.*, 2003).

### Ixodes ricinus collection and feeding success

*Ixodes ricinus* nymphs were caught by dragging a white flannel flag over suitable vegetation. The ticks were subsequently kept under sterile conditions in a climate room at >90% relative humidity, a 16 h:8 h (light:dark) photoperiod, and a 25°C:15°C temperature cycle until infestation. After feeding on the blue tits, the engorged nymphs were weighed to the nearest 0.01 mg. To investigate the influence of repeated infestations on feeding success, we estimated the following parameters: (1) the proportion of the administered ticks that successfully engorged (tick yield), (2) the total weight of engorged nymphs and (3) their feeding duration. If hosts acquire resistance, this is expected to result in the following observations compared to naïve hosts ( Rechav, 1992): lower numbers of engorged ticks, smaller blood meals (lower weight of engorged ticks), and increased feeding durations. From these criteria, engorgement weight is considered as to be one of the most consistent indicators of resistance ( Varma *et al.*, 1990).

### ELISA-based detection of immunoglobulins

***Tick’s salivary gland extraction (SGE).*** Salivary glands were dissected from 32 semi-fed colony-reared infection-free adult female *I. ricinus* obtained from IS insect Services GmbH in Berlin, Germany. Before dissection, all ticks had fed on sheep for 5–7 days; feeding is known to significantly increase SGE concentrations ( Mateos-Hernández *et al.*, 2017; Ogden *et al.*, 2002). The glands were washed four times in PBS to remove tick debris, pooled and homogenized. To test IgY-cross-reactivity with antigens of a congeneric tick species, we used material of 12 engorged adults *I. arboricola* ticks ( Heylen *et al.*, 2014) that had fed on great tit nestlings and that were dissected in the same way. A pool, containing the glands of 6–8 ticks in 60 μl PBS, was manually disrupted with a sterile pestle and the following steps were performed before storage at -80°C: sonication three times for five seconds with a treatment in ice (BRANSON 150), centrifugation at the maximum speed (10,000 rpm) for 10 minutes at 4°C, and filtering through a 0.2 μm filter (Chromafil AO-20/3 Macherey-Nagel GmbH, Düren, Germany) to remove contaminating bacteria. Total protein concentrations were estimated by Nanodrop ( Cafiso *et al.*, 2019) and equilibrated to 1 mg/mL prior to use in the assays.

***IgY ELISA.*** Basing ourselves on Ogden *et al.* (2002), after optimization of the ELISA-protocol - including the binding capacities of the anti-chicken antibodies for passerine raised antibodies (sandwich ELISA in which plates are coated with bird sera) – the following volumes and concentrations yielded the most reliable and repeatable results for the indirect ELISA tests. To coat the 96-well microtiter plates (Nunc Maxisorp flat bottom, Thermo Fisher Scientific, Geel, Belgium), 150 µL PBS per well was used, containing a concentration of 1.8 μg/mL SGE. Negative controls were coated with 150 μL PBS only. Plates were incubated for 12 hours at 4°C. After coating, the plates were washed three times with 200 mL PBS to remove the unbound material prior to blocking, using 200 µL of 0.5% Bovine Serum Albumin in PBS per well, and incubation for 1h at 37°C. Subsequently, the solution was removed and the wells were rinsed with PBS. Primary antibodies were obtained from bird sera diluted 140 times in PBS. 150 µL of the diluted bird sera were added to appropriate wells and the plates were incubated for 1 hour at 37°C. Afterwards, the wells were washed four times with 200 mL of PBS and 150 µL of the labelled secondary antibody (Rabbit anti-Chicken IgG, FC specific-alkaline phosphatase antibody, Sigma-Aldrich, code SAB3700239, Overijse, Belgium -15000 times diluted in PBS) was added. After one hour incubation at 37°C, the plates were washed three times with 200 mL of PBS and pre-washed once with 200 mL of alkaline phosphatase (AP) buffer (100 mM Tris, 2 mM MgCl2, pH 9.6 with HCl). The amount of secondary antibody bound to the primary antibodies is visualized through AP reaction after adding 150 µL of a 1 mg/mL 4- *p*-nitrofenylfosfaat dilution in AP reaction buffer and one hour incubation at 37°C. Plates are read with a plate reader (Biotek Synergy MX, BioTek, Winooski, VT, USA) measuring OD at 405nm.

***Repeatability and qualitative discrimination infested vs. non-infested birds.*** Negative control serum samples were obtained from three adult (sex unknown) domesticated canaries ( *Serinus canaria*, L. 1758), belonging to a captive population maintained for multiple generations (15 years) at the University of Antwerp. In addition, three 1 ^st^ calendar year blue tits ( *Cyanistes caeruleus*) and three 1 ^st^ calendar year great tits ( *Parus major*, L. 1758) that were kept in tick-free aviaries since hatching (sex unknown; see Heylen *et al.*, 2010 for further details on origin and housing). Sera from tick-exposed birds were obtained from three free-living great tits (1 ^st^ calendar male and female, one 2 ^nd^ calendar year male) that showed to be *Ixodes ricinus* tick-infested upon capture with mist nets (early Autumn 2019, Antwerp, Belgium), three 1 ^st^ calendar year great tits (sex unknown) that were three times experimentally exposed to 17 *Ixodes ricinus* nymphs over a time span of 30 days ( Heylen *et al.*, 2010), and three of the abovementioned blue tits.

**Figure 2.**
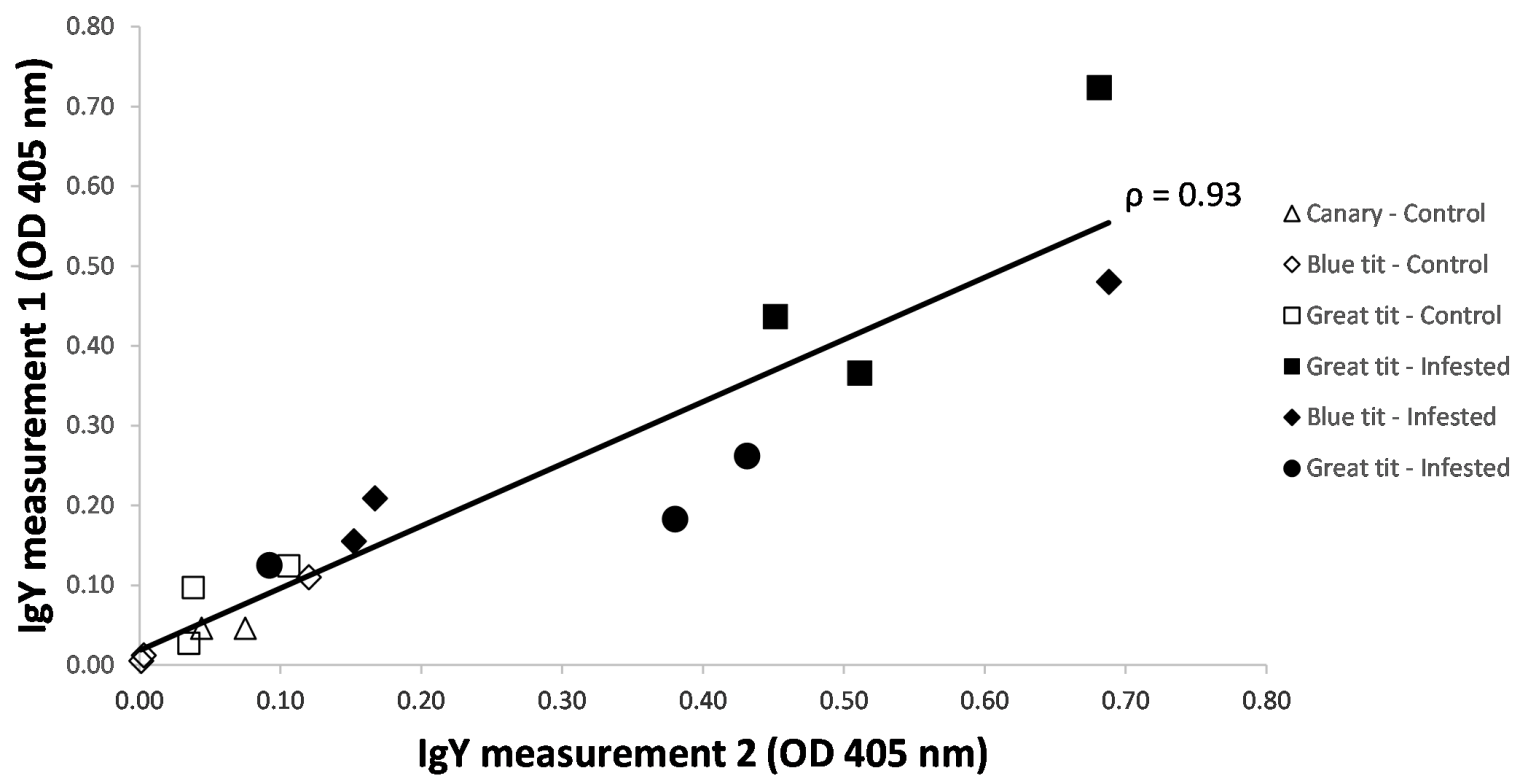
ELISA’s optical density measures of two IgY-measurements on the same bird. IgY levels were obtained from non-infested and *Ixodes ricinus*-infested birds (great tit, blue tit, canary). Among the infested birds, three great tits were very recently infested when caught in the wild (filled squares). All other samples were obtained from birds that fledged in tick-free aviaries or cages (control), and which were three times experimentally infested with ticks (including bird 1–3 of the 16 birds in the blue tit experiment).

The IgY levels in serum samples belonging to the same individuals showed to be highly repeatable within an ELISA-plate (Pearson’s Rho: 0.93; N= 17) as depicted by the scatterplot ( Figure 2). One measurement was excluded (in the non-infested Sc 3) as a pipetting error had occurred. Considerable variation in IgY levels was observed among tick-exposed individuals of the same species (variance/mean in great tits: 10%; blue tits: 19%), but also in the naïve blue tits (10%). Both naturally infested (caught in the wild and blood sampled once) birds and repeatedly infested birds (following scheme depicted in Figure 1) showed noticeably higher OD values than non-infested individuals.

### Statistical analysis

**Figure 3.**
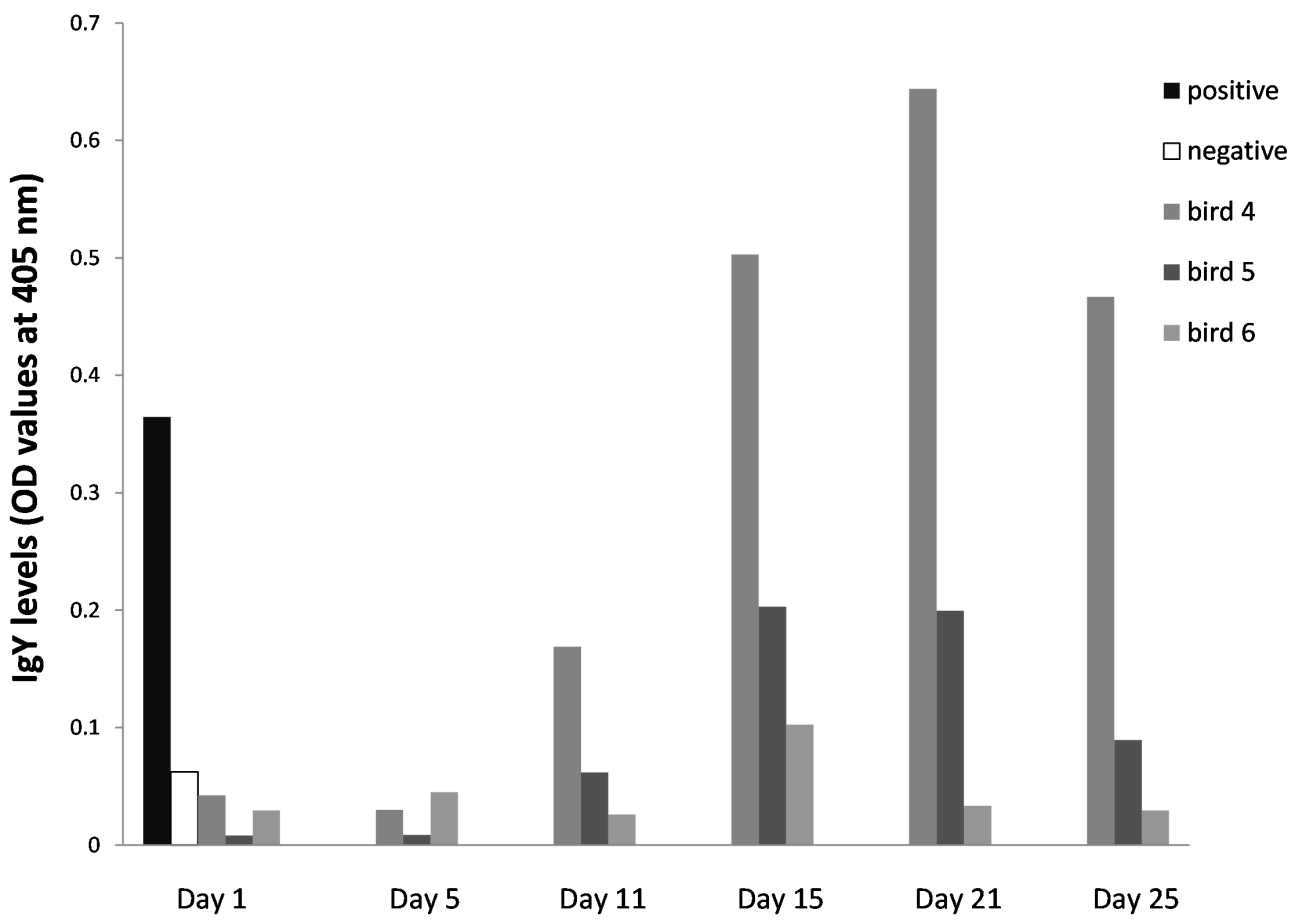
IgY levels of sera sampled from *Ixodes ricinus*-infested blue tit individuals (bird 4–6) that were repeatedly exposed following the study design in Figure 1 (pre-infestation: Day1, 11, 21; post-infestation: Day 5, 15 and 25). Positive and negative control birds: repeatedly exposed great tit and naïve canary, respectively.

For the qualitative comparisons between infested and non-infested birds (only three individuals per bird species, over the two groups) no statistical tests were performed ( Figure 2), neither for the description of the IgY-profiles of three blue tits - for which sufficient amounts of serum and tick antigens allowed a quantification at each of the six time points ( Figure 1 and Figure 3). Data of IgY levels at Day 1, 11 and 21 ( Figure 1) of the latter three birds were combined with that of ten additional blue tits, followed by the parametric statistical analyses as described below:

generalized linear mixed effect models (GLMM’s) were fitted on health measures to model the acute infestation effects (Δ Inf. 1, 2 and 3; Figure 1) as a function of the bird’s IgY levels (OD value) within each infestation. To avoid collinearity problems and to adjust for differences in variation between infestations, the IgY levels were standardized (OD _Inf.x_ - mean _Inf.x_/Standard deviation _Inf.x_). By adding a random bird individual effect, and using Kenward-Roger approximation for the denominator degrees of freedom, we took into account the correlation of observations within the same individuals.

In a second statistical analysis, we tested whether the changes in acute effects (‘Δ Acute’, Figure 1) were related to the summed IgY levels over the three infestations (SOD), which we consider as a proxy for the bird’s overall anti-tick IgY production over the course of the experiment. In a final analysis we modelled the change in initial values (Δ Inf. 3–1) (= chronic response) as a function of SOD. Effects of IgY levels on tick feeding parameters were modelled in a similar way, except for the fact that we only have one value per bird/infestation session (and not a difference). Before entering the analysis, the average tick measures (weight, feeding duration) were calculated for each bird/infestation. In all models, a stepwise selection procedure was used in which the model was iteratively refitted after exclusion of the least significant effect, until only significant factors and their lower order interactions terms were left. All data manipulations and statistical analyses were performed using SAS v 9.2 (SAS Institute, Cary, North Carolina, USA). Estimates are reported as mean ± standard error.

## Results

### Sera *I. ricinus*-exposed birds and cross-reactivity with *I. arboricola* antigens

**Figure 4.**
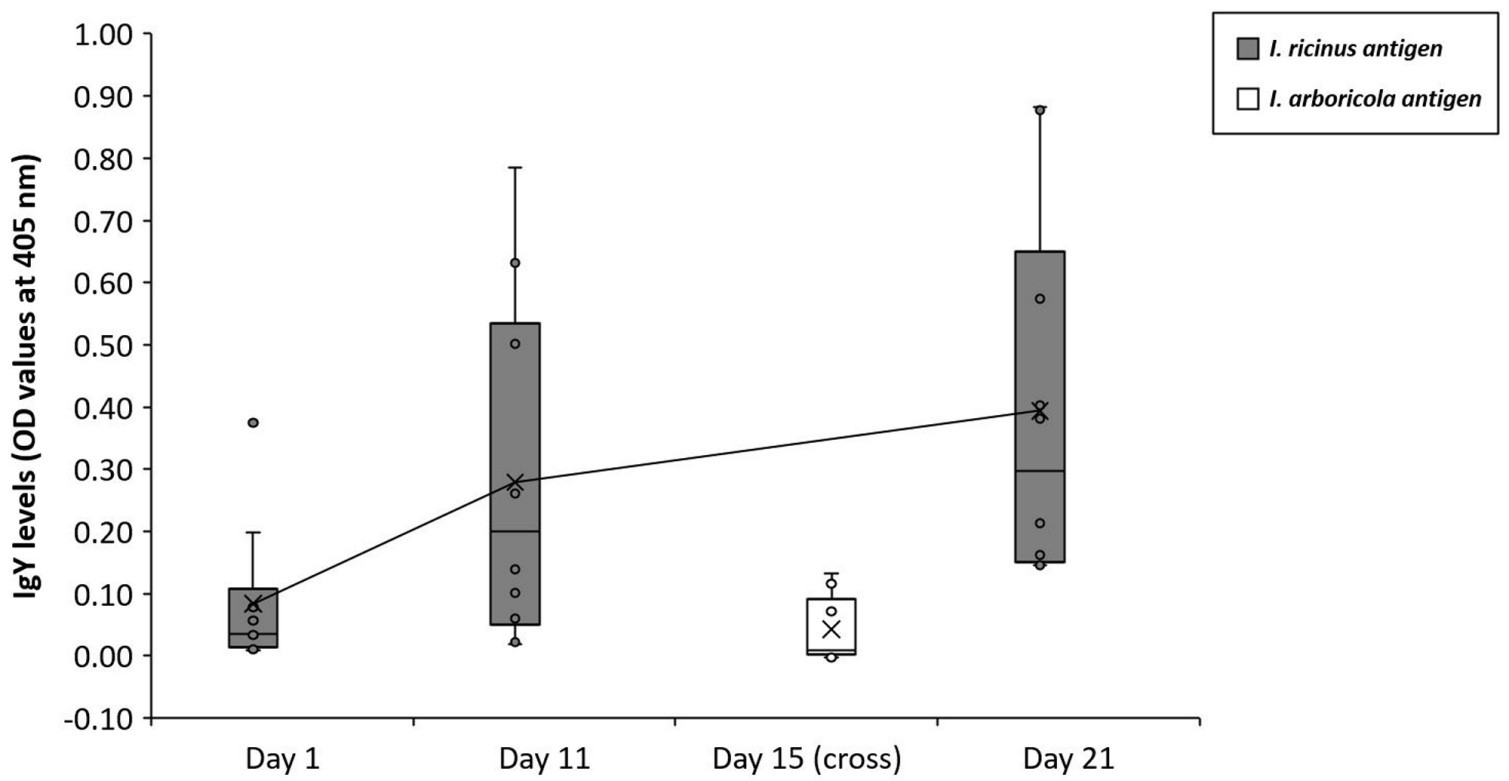
Box-plots of the IgY levels of sera from ten blue tits (bird 7–16) that were infested three times in succession with *I. ricinus* (for design see Figure 1). Values of a second ELISA are included, in which the cross-reactivity of anti- *I. ricinus* IgY’s was tested (Day 15 samples only) against *I. arboricola* salivary antigens.

In order to evaluate the entire temporal patterns in IgY levels over the course of the experiment, we monitored the three blue tit individuals for which sufficient serum was available at all possible sample occasions (i.e. six moments in total, see Figure 1 for study design). We observed an OD-curve that tended to be bell-shaped, with the highest IgY levels around Day 15–21 ( Figure 3). The same increase in temporal pattern (Day 1, Day 11 and Day 21) was observed in the additional ten blue tits (0.014 ± 0.004 OD unit/Day unit, T-value = 3.49, df = 10.8, P = 0.0052; Figure 4). Large individual variation was observed on each of the sampling days (variance/mean Day 1: 5%, Day 11: 17%, Day 21: 16%), and variation in slopes differed from zero (estimate: 0.14 ± 0.1 10 -3; Likelihood ratio test: Z = 1.70; P = 0.044). We repeat that the reasons why the ten birds are not entirely covered are: (1) at several sample occasions there was not enough serum available, (2) the Day 15 sample has been used to investigate cross-reactivity with *I. arboricola* antigens. With regard to the latter, the antibodies against *I. ricinus*-antigens in those samples taken at Day15 showed almost no cross-reactivity ( Figure 4). We observed that in two birds (bird 2 and 4) the OD’s in the *I. arboricola* wells were slightly higher than the baseline values on Day 1 (i.e. when all birds were still naïve.

### IgY correlations with anti-tick resistance

In order to investigate whether IgY influenced the tick’s feeding performance, we utilised a GLMM for repeated measurements with feeding performance (engorgement weights, feeding durations or engorgement success) as response variable and the IgY levels as explanatory variable.

**Figure 5.**
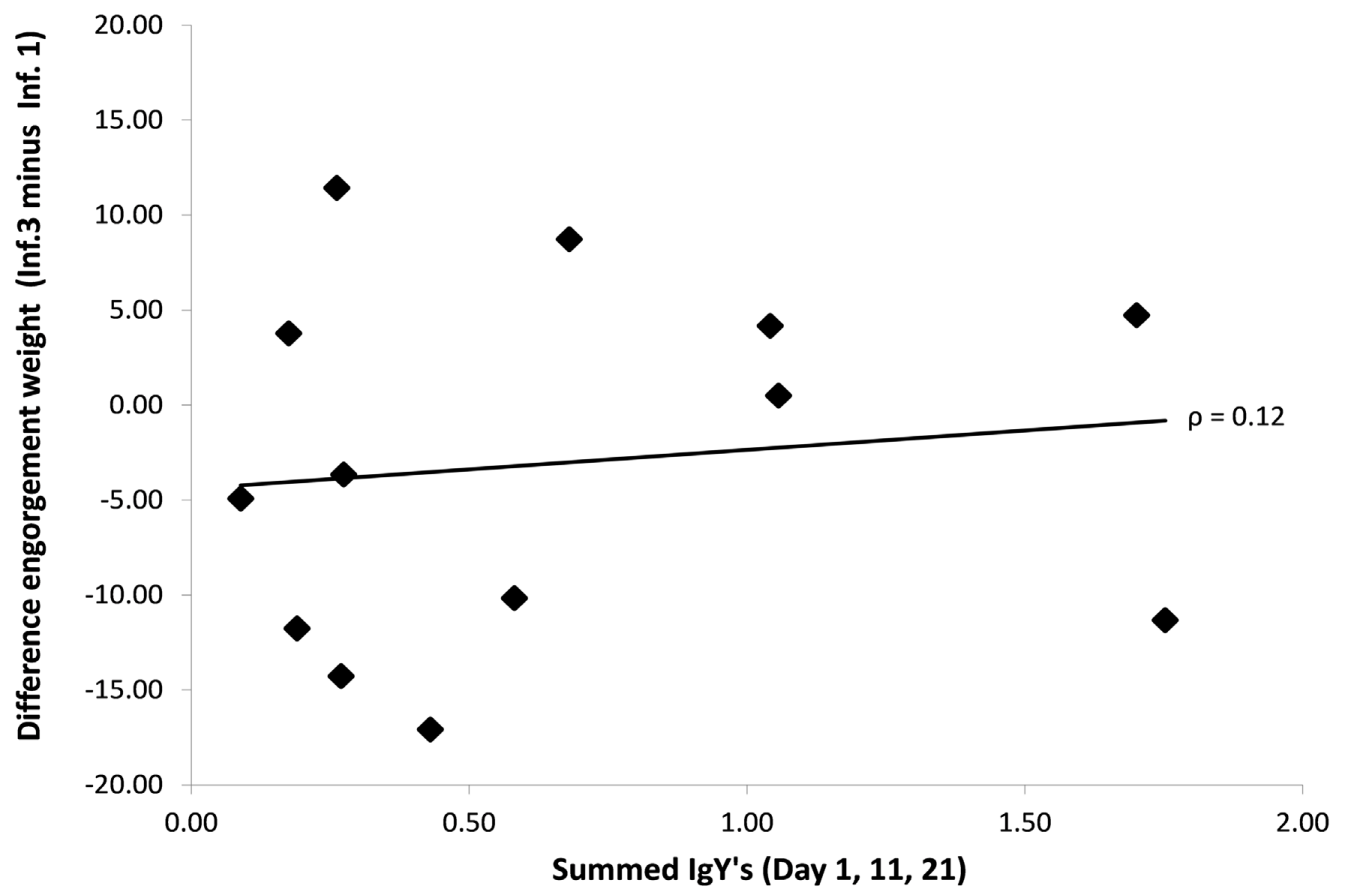
Difference in the summed engorgement weights between the beginning of the experiment (Inf. 1, naïve bird) and the end (Inf. 3, previously exposed to 24 ticks) as function of the summed IgY levels in blue tit sera (bird 4–16). Total engorgement weight is a function of the average engorgement weight and the proportion of successfully fed ticks, none of which showed a significant association with IgY levels ( Table 1).

Test statistics for the cross-sectional (Inf. 1, 2 and 3) and longitudinal analyses (Δ Inf. 3–Inf. 1) in relation to the IgY levels are presented in Table 1. Neither the average engorgement weight ( Figure 5) nor the feeding duration showed significant associations with IgY level in any of the analyses ( Table 1), despite the strong IgY-increase and large among-individual variation ( Figure 3 and Figure 4). Furthermore, the proportion of ticks that successfully engorged did not covary with IgY levels. For absolute values of feeding parameters, we refer to Heylen *et al.* (2010). Conclusions did not change when restricting the analyses to the subset of 10 individuals that were simultaneously analysed on a single plate ( Figure 4). In sum, none of the feeding performance factors were influenced by IgY levels across either each infestation, or across all three infections.

**Table 1.** Type 3-outcomes of generalized linear mixed effect models (GLMM's) investigating the association between IgY levels and proxies for anti-tick resistance. IgY levels were measured in the serum samples taken at the beginning of each infestation session. In the analyses of the cross-sectional correlations (‘per infestation’) IgY levels have been standardized. For the longitudinal analysis (i.e. the cumulative response, ‘Inf. 3 minus Inf. 1’), the summed IgY levels over the three infestation sessions was calculated (See Figure 1). Test statistics before exclusion from the model are given, as well as the parameter estimates and statistics for the terms that remained in the model (P-value <0.05).

*Per infestation*	*Infestation* _(ndf,ddf)_ *F*	*IgY (SD)* _(ndf,ddf)_ *F*	*IGY x Infestation* _(ndf,ddf)_ *F*
*Engorgement weight*	_(1,35)_0.15 ^NS^	_(1,37)_0.21 ^NS^	_(2,33)_0.56 ^NS^
*Feeding duration*	_(2,35)_0.74 ^NS^	_(1,37)_2.74 ^NS^	_(2,33)_1.20 ^NS^
*Engorgement success*	_(2,31)_0.63 ^NS^	_(1,36)_0.64 ^NS^	_(2,32)_0.62 ^NS^
			
***Inf. 3 minus Inf. 1***		***Summed IgY’s*** **_(ndf,ddf)_ *F***	
*Engorgement weight*		_(1,11)_1.81 ^NS^	
*Feeding duration*		_(1,11)_0.55 ^NS^	
*Engorgement success*		_(1,11)_0.70 ^NS^	

Infestation: early (Inf. 1), during sero-conversion (Inf. 2) and at maximum IgY levels (Inf. 3); NS: P-value >0.05.

### IgY correlations with tick virulence

**Figure 6.**
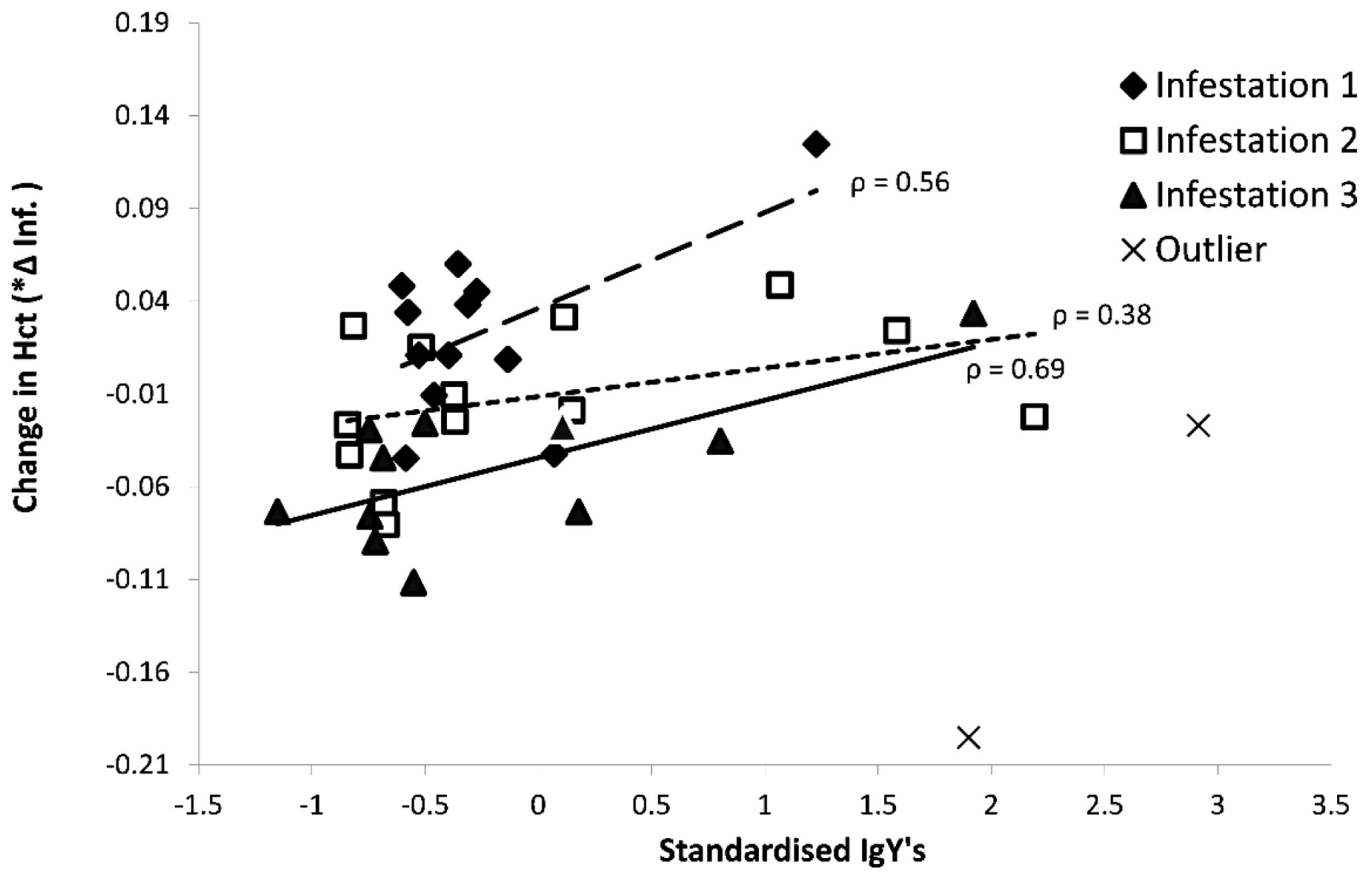
Acute effects of ticks on Hct levels in response to the standardized IgY levels of sera samples in 13 blue tits (bird 4-16) presented in Figure 3 and Figure 4. Two outliers from the same individual were excluded from the statistical analyses.

In order the assess the influence of IgY on the acute health effects by the feeding ticks (i.e. the birds’ changes in Hct and body condition), we used a GLMM with as response variable the acute change in the health parameter within an infestation session (see Figure 1), and as explanatory variable the IgY levels from the blood sample taken immediately before an infestation ( Table 2). While on average the Hct levels did not significantly change during the first two infestations (‘Acute’ Inf. 1: 1.97 ± 1.29; Inf. 2: -1.14 ± 1.08%), they decreased in the third infestation (Inf. 3: -6.24 ± 1.63%, T-value = -3.82, P = 0.0028). Birds with higher IgY levels prior to an infestation showed a less severe Hct decrease (2.23 ± 0.80%/OD unit, T-value = 2.92, df = 31, P = 0.0065; Figure 6). In this analysis, two statistical outliers belonging to the same bird were removed (see Figure 6). The same analysis, but with body condition as response variable, did not show any significant associations with IgY levels.

**Table 2.** Outcomes of GLMMs investigating the association between IgY levels and the health parameters in 13 birds repeatedly infested with *Ixodes ricinus* nymphs. See Figure 1 for the experimental design, and further explanation of ‘Acute effect’ (‘after’ minus ‘before’ exposure), Δ Acute (Change in acute effects Inf. 3 vs. Inf. 1) and Δ Chronic (values on Day 21 minus the ones on Day 1).

* Acute effect (Δ Inf.x)	*Infestation*	*IgY (SD)*	*IgY x Infestation*	*Average Slope over* * the 3 infestations*
*Haematocrit*	**_(2,31)_ **12.06** ^<0.001^**	**_(1,31)_ **8.76** ^0.006^**	_(2,29)_ 1.70 ^NS^	**0.024±0.008**
*Condition*	_(2,36)_ 0.98 ^NS^	_(1,35)_ 0.06 ^NS^	_(2,33)_ 0.69 ^NS^	
				
***^$^ Change in acute effect (Δ Acute)***		***Summed IgY’s*** **_*(ndf,ddf)*_ *F***		
*Haematocrit*		_(1,11)_0.16 ^NS^		
*Condition*		_(1,11)_4.42 ^0.064^		
				
***^£^ Chronic effect (Δ Chronic)***		***Summed IgY’s*** **_*(ndf,ddf)*_ *F***		***Slope***
*Haematocrit*		_(1,11)_ 0.65 ^NS^		
*Condition*		**_(1,11)_ **28.41** ^0.0002^**		**0.033±0.006**

Infestation: early (Inf. 1), during sero-conversion (Inf. 2) and at maximum IgY levels (Inf. 3).

* Outcome without the bird giving outlying residuals. This bird led to violation of the normality assumption (Shapiro-Wilk W: 0.96); removing it improved the model fit (Shapiro-Wilk W: 0.98).

NS: P-value >0.05.

When investigating the change in acute effects on Hct (‘Δ Acute’) caused by the ticks (i.e. the difference between the acute effect in infestation 1 and the one in infestation 3) we did not find an association with the total amount of IgY produced by the bird during the experiment (i.e. the summed IgY levels as a proxy). The same analysis, but with body condition as response variable, did not reveal any significant association with the summed IgY levels ( Table 2).

**Figure 7.**
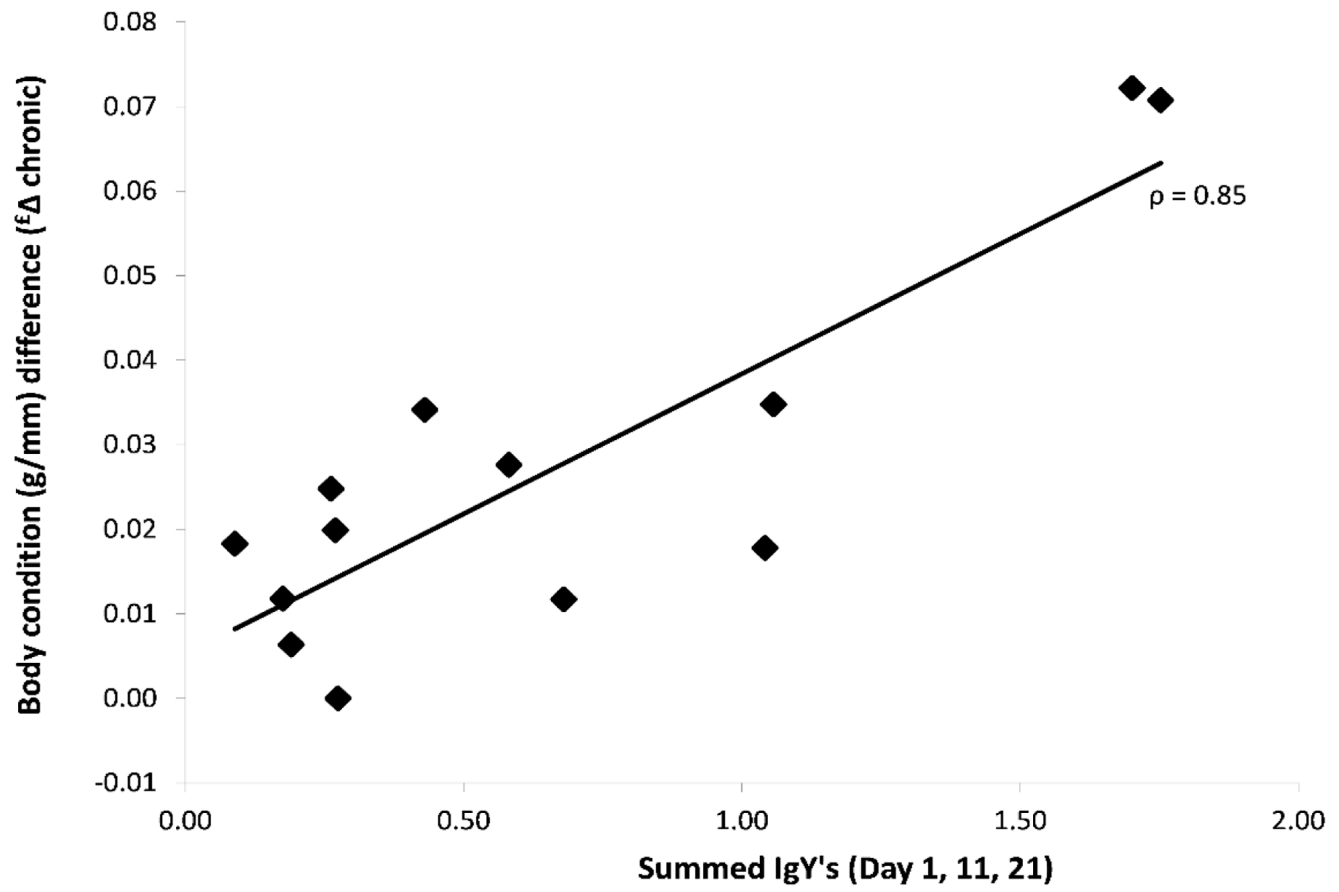
Chronic effects of ticks on the body condition (weight/tarsus) of 13 blue tits (bird 4-16) in response to the summed IgY levels (summed OD’s over three time points prior to an infestation).

In order the assess the influence of IgY on the chronic health effects by the ticks, we modelled the change in health (i.e. Day 21 minus the baseline values on Day 1, see Figure 1: Δ Chronic) against the summed IgY levels. We found that the increase in body condition over the infestation sessions (Δ Chronic: 0.027 ± 0.006 g/mm; T-value: 4.38, df = 11; P < 0.001) was higher in birds that had higher summed IgY levels (SOD) (0.033 ± 0.006 g/mm; T-value = 5.33, df = 11, P = 0.0002; Figure 7), however Hct levels did not show any association with SOD ( Table 2).

## Discussion

This is the first study that investigates the interplay between the songbirds’ humoral immune response and natural blood-sucking ectoparasites (ticks), combining observational and experimental approaches. The acquisition of tick immunity has been an important topic in the field of tick biology, as patterns of immunity can be used to develop transmission blocking vaccines for humans or reservoir animals against tickborne pathogens. Immunity of mammals and non-mammalian hosts (such as birds) may differ in response to the same antigens (e.g. tick salivary proteins), which emphasizes the need of the current study. Furthermore, as wild birds can maintain and spread tick-borne pathogens, the relevance of the use of wild phenotype birds for endemic transmission cycles likely outweighs the limitation linked to the maintenance of wild animals in lab conditions. We put forward three questions (1) do birds develop a specific antibody response against *Ixodes ricinus* salivary antigens and how strong is the variation among birds? (2) Does the humoral immune response reduce tick feeding success (3) and virulence?

In order to address the first question, we developed an indirect ELISA, and succeeded to quantify the bird’s immunoglobulins (IgY) that bind to *I. ricinus* salivary gland antigens. We then could show that the level of tick-specific IgY was low at the beginning of the experiment, when birds were naïve, then steeply increased to peak at 15–20 days - the moment of sero-conversion – and tended to decrease afterwards ( Figure 3). This sero-conversion pattern is comparable to IgG-kinetics in mammals ( Barriga *et al.*, 1991). IgG is the mammalian analogue to the avian IgY, present in the chronic phase of parasite exposure, and is involved in the development of long-term resistance ( Davison *et al.*, 2008) against ticks ( Ogden *et al.*, 2002). In our study, the IgY-response turned out to be specifically targeted against *I. ricinus* salivary antigens, as the cross-reactivity against *I. arboricola*-antigens was shown to be negligibly low ( Figure 4). Thus, the observed IgY-response gives evidence for an adaptive response, rather than an acute inflammatory reaction. We found significant individual variation among birds in immune profiles, as well as in initial values before being exposed for the first time. An explanation for the latter finding might be that maternal antibodies of mothers with anti-tick IgY-concentrations in their blood have been transferred via egg yolk to nestlings, as observed in gulls ( Müller *et al.*, 2004). This additional source of antibodies complicates the interpretation of IgY levels as signals of the individual’s previous tick exposure, especially in juveniles ( Gasparini *et al.*, 2001). However, the immunological processes in the early development of altricial birds (among which blue and great tits) differ from those of semi-precocial birds (to which gulls belong). In an experimental study by King *et al.* (2010) where House sparrows ( *Passer domesticus* L.) are experimentally exposed to novel antigens, the authors observed short half-life of maternal antibodies (less than 3 days after hatching), low transfer from mother to nestlings, and a rapid production of endogenous antibodies by nestlings (8–10 days after hatching). They concluded that altricial developing birds achieve immunologic independence much earlier than precocial birds, implying that the variation in (initial) IgY levels observed in our study is synthesized by the juvenile bird itself.

For our second question, we looked at pairwise relationships between the IgY levels and either tick feeding parameters or bird health measures. As observed in other natural hosts ( Fielden *et al.*, 1992; Ribeiro, 1989), the opportunistic *I. ricinus* turns out to be extremely efficient in circumventing the bird’s antibody response. In host types where anti-tick resistance is acquired, strong decreases in engorgement weights are observed in subsequent tick exposures. Acquired resistance against ticks has frequently been demonstrated in laboratory rather than in natural hosts, therefore it has been suggested that tick resistance is confined to artificial host–tick associations (e.g. guinea pigs infested with Dermacentor variabilis, rabbits infested with *I. ricinus*) ( Bowessidjaou *et al.*, 1977; Ribeiro, 1989; Wikel, 1996) and that successful parasitism in natural host-tick associations is the result of an intense co-evolution, in which ticks developed adaptations to evade the host’s immune system ( Fielden *et al.*, 1992; Ribeiro, 1989). Here we found that, despite the high among bird-individual variation in IgY-responses and tick feeding success, there was no significant association between them. We conclude that naïve juveniles do not acquire immunological anti-tick resistance. The outcomes could be viewed as a mechanism of tolerance instead of resistance. The latter is the capacity to limit the parasite burden, while the first refers to the ability to limit the harm caused by a given burden by compensatory mechanisms ( Råberg *et al.*, 2009). The concept of tolerance is notoriously difficult to measure in animals, when measuring the slope of how host fitness decreases with parasite burden. Here, high burdens did not cause direct fitness effects (bird mortality), or gave rise to indirect fitness effects via physiological measure that link up with bird fitness; both findings are in favour of tolerance.

While on average the ticks performed equally well throughout the experiment (i.e. no significant difference between infestation 3 and 1), the harm (i.e. blood depletion) seemed to be better compensated for when birds had higher IgY levels. Massive amounts of reticulocytes (i.e. immature erythrocytes) are stored in bird bone marrow, and can be instantly released in the blood stream ( Martinho, 2012). We point out that the net Hct difference in each infestation ( Figure 1 – Figure 6) is the result of two processes: the immediate erythrocyte compensation (i.e. addition of erythrocytes in the blood stream) and acute erythrocyte depletion due to tick feeding. Although tick feeding did not decrease with IgY, the net effect of the abovementioned processes showed a correlation with IgY. We do not know of any role of IgY in these processes, but since both immune responses and harm compensation are energetically demanding, general health could simply be driving the observed correlation. Additionally, birds with a higher overall IgY-response gained more body weight (first 21 days). Metabolic processes for the compensation of the blood depletion by the ticks, the repair of skin lesions and blood vessels, and mounting immune responses are all energy demanding ( Martin *et al.*, 2003), and may lead to a reduced body condition. However, under lab conditions, with food *ad libitum*, those birds with the strongest IgY-response showed to be more successful in gaining body mass. The observed increase may relate to the gain in body mass for the regeneration of feathers (i.e. the post-juvenile moult) ( Bojarinova *et al.*, 1999) or other undefined seasonal physiological changes. Also, by gaining body mass, birds possibly anticipated the costs of (chronic) activation of the immune system due to tick infestations and/or ongoing tick-borne pathogen infections (as birds were exposed to ticks from the wild) ( Heylen *et al.*, 2015). In the end, this may benefit the fitness of both the ticks and micro-organism: future ticks could feed more successfully in those birds with a stronger body mass increase and it is conceivable that they may even induce such processes. We mention that our results are correlational, and do not prove causation; anti-tick IgY-response are not necessarily the cause of better health outcomes, but could be a correlated by-product of variation in quality among the birds. This quality (condition, health, vigour) could be affected by several factors, including good genetic constitution, higher quality maternal care, lower stress experience, fewer co-parasites, etc. In the ecological immunology literature, many studies have shown that variation in condition will drive associations between immunological traits ( Sadd & Schmid-Hempel, 2009) but from our study it is clear that the measured IgY’s did not correlate with the tick’s feeding success, despite they targeted tick salivary antigens.

Birds are central elements in the ecological networks of ticks, but are heavily overlooked when it comes to elementary biological mechanisms like immune responses ( De la Fuente *et al.*, 2015). In fact, surprisingly few studies have related individual variation in host immunity to components of ectoparasite or even tick- fitness. Despite being unsuccessful in reducing tick feeding success via IgY’s, birds may benefit from the observed IgY-responses: by indirectly acting against vector-borne pathogen constituents (i.e. tick proteins functioning as carrier vehicles) tick-to-host transmission may become mitigated. As the transmission of pathogenic tick-borne agents heavily relies on salivary proteins ( De la Fuente *et al.*, 2015), it is worth studying this hypothesis for a variety of tick-borne pathogens in (in)competent natural reservoir songbird hosts. Are anti-tick immune responses, host physiology and tick feeding performance affected by the pathogens themselves and/or other tick associated micro-organisms (including viruses)? Are these responses comparable in different tick-pathogen-host systems? Does also the bird’s long-term fitness remain unaffected by high tick loads, providing further indirect evidence for tolerance (as defined by Råberg & colleagues (2009))? All these questions are heavily unexplored in birds, but are crucial for understanding local transmission and life cycles. Some of the answers could even inspire vaccine development, when mapping the tick epitopes that are effectively used by pathogens and could be targeted by host immune components.

## Data availability

Zenodo: data Ineffective humoral anti-tick IgY-response in birds reaction against pathogen constituents, https://doi.org/10.5281/zenodo.4527196 ( Heylen, 2021).

Data are available under the terms of the Creative Commons Attribution 4.0 International license (CC-BY 4.0).
