# Supplementary material for: Ineffective humoral anti-tick IgY-response in birds: reaction against pathogen constituents?
Source: Open Res Eur. 2021 Sep 23;1:8. Originally published 2021 Mar 24. [Version 2] doi: 10.12688/openreseurope.13204.2 (PMC10446090; doi:10.12688/openreseurope.13204.2)
Supplement: Peer review history

# Peer review: Reviewer response for version 2

**DOI:** 10.21956/openreseurope.15184.r28390

**Published:** 2022-02-07

**Authors:** Courtney Waugh

**Affiliations:** 1 Faculty of Biosciences and Aquaculture, Nord University, Bodø, Norway

This article provides new and important information about the humoral immune response of blue tits and their ticks.

 The work is clearly presented in adequate detail and cites relevant sources.  The figures are clear and the statistics are sound. The study is well designed to answer the research questions and provides new data about the subject. The conclusions support the results.

 A very nice paper.

Is the study design appropriate and does the work have academic merit?

Yes

Is the work clearly and accurately presented and does it cite the current literature?

Yes

If applicable, is the statistical analysis and its interpretation appropriate?

Yes

Are all the source data underlying the results available to ensure full reproducibility?

Yes

Are the conclusions drawn adequately supported by the results?

Yes

Are sufficient details of methods and analysis provided to allow replication by others?

Yes

Reviewer Expertise:

Wildlife immunology and infectious diseases.

I confirm that I have read this submission and believe that I have an appropriate level of expertise to confirm that it is of an acceptable scientific standard.

# Peer review: Reviewer response for version 2

**DOI:** 10.21956/openreseurope.15184.r28391

**Published:** 2022-02-03

**Authors:** Veerle Jaspers

**Affiliations:** 1 Department of Biology, Norwegian University of Science and Technology (NTNU), Trondheim, Norway

The authors present an original contribution regarding the immunological responses of birds to ectoparasite infections and the potential coping mechanisms of the birds. The results are very interesting and the discussion is sound and highlights areas for further research.

 After reading the revised manuscript and the responses the authors have made regarding the comments of earlier reviewers, I am of the opinion that the manuscript is suitable to pass peer review but would suggest a few minor revisions.

 I have some minor comments in addition to the revisions already made. All are regarding clarification of the methods:

- Please add the number of blue tits exposed for this experiment in the methods section of the abstract.
- In my opinion, it would be clearer for the reader to move the section on Ixodes ricinus collection up, just after the ethical statement.
- One clarification is needed: I got confused about the number of birds for each specific result: 16 blue tits in total were used, but for only 3 enough blood was available at 6 times. Then again the authors talk about 10 additional birds. So these two together account for 13 birds. What happened to the other three? I got confused here and it would be helpful to list specifically in the statistical part of the methods and also clearly in the results, how the 16 birds were included/divided in the different parts of the analysis.

Is the study design appropriate and does the work have academic merit?

Yes

Is the work clearly and accurately presented and does it cite the current literature?

Yes

If applicable, is the statistical analysis and its interpretation appropriate?

Yes

Are all the source data underlying the results available to ensure full reproducibility?

Yes

Are the conclusions drawn adequately supported by the results?

Yes

Are sufficient details of methods and analysis provided to allow replication by others?

Yes

Reviewer Expertise:

Avian toxicology, ecotoxicology, avian physiology

I confirm that I have read this submission and believe that I have an appropriate level of expertise to confirm that it is of an acceptable scientific standard.

# Peer review: Reviewer response for version 1

**DOI:** 10.21956/openreseurope.14274.r26998

**Published:** 2021-08-20

**Authors:** Maxime Madder

**Affiliations:** 1 The Tamarin Commercial Hub, Clinglobal, Tamarin, Mauritius

**Ineffective humoral anti-tick IgY-response in birds: reaction against pathogen constituents. Heyler *et al*.**

 The authors present an interesting study trying to define the effect of a humoral response in blue tits that are exposed to multiple infestations with wild caught I *xodes ricinus* ticks.

**General comments**

 The paper offers an interesting insight the way blue ticks react to multiple infestations of ticks, but some general information is missing as pointed out below.

**Introduction**

 It might be interesting for the reader to explain what type of host immune responses are triggered by ticks (including birds), as other studies were referred to (Heylen et al., 2010, 2015), and this to explain why IgY was selected. Also why IgY was looked at and not IgA or M.

**Methods**

 Having used wild caught ticks, and not knowing their infection status with viral, bacterial or protozoal disease agents, could the authors clarify if the use of infected ticks might have influenced the results and conclusions? Many articles describe the influence of infected ticks on the ticks' behaviour, but also on the host immune response.

 Could the authors specify where the ticks were collected, what the known pathogens are in this region and their prevalence, and where the birds originate from. Also the date the study was conducted is not specified.

**Discussion**

 The authors state the the birds tolerated the ticks, and did not show anti-tick resistance. Could this be dose-dependent? Could higher infestation loads result in anti-tick resistance? It might be hypothesised that tolerance is seen as long as birds are not negatively impacted by infestation.

Is the study design appropriate and does the work have academic merit?

Yes

Is the work clearly and accurately presented and does it cite the current literature?

Yes

If applicable, is the statistical analysis and its interpretation appropriate?

Yes

Are all the source data underlying the results available to ensure full reproducibility?

Yes

Are the conclusions drawn adequately supported by the results?

Yes

Are sufficient details of methods and analysis provided to allow replication by others?

Yes

Reviewer Expertise:

Parasitology, acarology, ticks and tick-borne diseases, vector ecology, animal health

I confirm that I have read this submission and believe that I have an appropriate level of expertise to confirm that it is of an acceptable scientific standard.

# Peer review: Reviewer response for version 1

**DOI:** 10.21956/openreseurope.14274.r27346

**Published:** 2021-08-10

**Authors:** Michelle Wille

**Affiliations:** 1 University of Sydney, Sydney, NSW, Australia

**Heylen: Ineffective humoral anti-tick IgY-response in birds: reaction against pathogen constituents**

 The authors aimed to understand the immune response of Blue tits to tick infestation using an experimental approach. Overall, this is an interesting paper, but currently some shortcomings in data presentation (both text and writing) make it hard to understand the details and interpret of the findings.

**Some general questions:**

 I think a bit more careful explanation is needed for the reader to understand the idea of “tick resistance”. Specifically, that it's not the prevention of ticks infesting birds, but rather a decrease in the impact of ticks on birds (eg, lower tick engorgements and perhaps a more effective immune response).

 Second, humoral immunity develops following the acute response (inflammation). How do these findings link into studies assessing inflammation following tick infestation? Would this variation in IgY perhaps be due to a variation in acute responses? I recognise that the authors did not measure this, but I wonder if there is data from previous studies?

 Third, as these were field collected ticks, how do the authors control for things like viral infections which may affect Hct or body condition. These viral infections may also affect Engorgement weights and feeding durations of ticks (particularly for viruses infecting the ticks, not necessarily arboviruses which are transmitted to the birds). Theoretically they shouldn’t affect the IgY as this is specific. I recognise that the authors did not measure this, but it is worth point out in the discussion.

**Specific comments:**

**Methods**

- I would defer to the expertise of other reviewers to appropriately assess the methods of this paper.

**Results**

*“Sera I. Ricinus-exposed birds and…”:*

- I would encourage the authors to open the first sentence with the purpose of this experiment. For example, “In order to address XXXX, we three tits were monitored at six time points (as opposed to 3). In these birds we found….”
- Similarly for the next paragraph. “Following this, 10 blue tits (including or excluding the 3 before?) were monitored at three time points. These time points corresponded to direction prior to infestation, and thus reflecting the IgY levels following the previous infestation event. In these birds, OD profiles were….”

*“IgY correlations with anti tick resistance”:*

- The opening of the second paragraph could improve. Something like: “In order to investigate whether XX influenced YY, we utilised a GLMM. Neither age etc...”
- Also, please help the reader a bit more. I’m not sure why “Inf 1” is cross-sectional and “delta Inf3-Inf 1" is longitudinal. Can you explain here what these values mean (especially given Figure 1 is not at all intuitive). So, based on this analysis, what did influence IgY levels? Perhaps add, “In sum, none of the factors we tested influenced IgY levels across either each infestation, or across all three infections”.

" *IgY correlations with tick virulence”:*

- This section is not well written – its rather written like a list of bullet points rather than a “story” whereby the reader is walked through the analysis with care. For example, adding leader statements like “In order the assess the acute effects, i.e., the variation in Hct levels, XXX YY” are useful.
- Why were these data points deemed outliers – was this a technical problem?
- Why were the IgY levels summed?

*RE Body condition:*

- Perhaps rephrase to say something like “Body condition...for all birds, with the exception of infestation sessions wherein we found an acute effect (here explain which sessions those were).” I also don’t understand why there is reference to chronic effect here?

*Paragraph 4:*

- Does this mean that you did the analysis twice – once including and once excluding the Birds in Figure 3 (i.e., the birds that were measured 6 times and therefore did not have enough sera remaining to be on all plates)?

*Paragraph 5:*

- Again, more common language interpretations would be useful here. Why did you use summed values? Why does Hct and Body condition refer to chronic? See comments above about including a leading sentence.

**Discussion**

*Paragraph 2 RE maternal antibodies:*

- Are there studies you can reference that have quantified how long maternal antibodies persist in birds. For example, this has been done in ducks for antibodies against influenza A, but I wonder if this has been done for any passerines for any virus/bacteria? This data is useful for future studies such that you may start experiments when birds are older, and you are sure that the effect of maternal antibodies has waned.

*Paragraph 3 RE tick resistance:*

- Can the authors please provide an example of hosts types that have been shown to acquire resistance to ticks?
- Also, you have a statement: “We conclude that naïve juveniles do not acquire anti-tick resistance”. Do adult passerines acquire this resistance? Are you sure you are using the correct measurements and experimental design for this as it is a strong statement to make?

*Paragraph 3 RE tolerance.*

- Given fitness wasn’t really measured and the experimental design limited and controlled for tick infestations, I am not sure how convincing this is. Are there are examples that the authors could reference for animals that tolerate ticks?

**My suggestions for Figure improvement:**

 I would think that a number of the figures that are related could be panelled into a larger figure? For example, Fig 3 and 4 together, perhaps Fig 5, 6, 7 together?

*Figure 1:*

- Concept figures should be immediately understandable without needing to read the legend, but unfortunately Figure 1 is very confusing. There is plenty of space so I suggestion you write out “Inf. 1” and “D1”. The legend isn’t very clear either. The figure doesn’t really explain well the different between “Acute” and “Chronic”. Overall, I suggest reconsidering this figure – there are lots of great concept figures out there in papers that rely on infection experiments for inspiration.

*Figure 2:*

- Based on the Figure and the legend, it is unclear to me the purpose of this figure. Perhaps a more descriptive title such as: Correlation between first and second IgY measurement of the same sample/bird”. Can you please add the Statistic onto the figure, and also the line. I expect that you could leverage more colours to make it easier for the reader.

*Figure 3:*

- Again, I would change the figure legend so that this plot is easier to appreciate. For example: Change in IgY levels of 3 Blue Tits that were sampled on 6 occasions. Given Figure 1 isn't intuitive, I still don’t really understand this plot. Perhaps add some extra metadata below the bars – lines or square brackets to indicate infestation periods, or different colours or arrows? Were the positive and negatives only for Day 1?

*Figure 4.:*

- I would suggest that this plot be a boxplot or point graph, with day on the x-axis, and the different birds in different colours.
- You could, if you wanted, connect the individuals with lines. You could also then add a line or similar for the mean? Even better, you could model the change of IgY over time? An example of a suggestion: https://stackoverflow.com/questions/59693411/r-boxplot-draw-lines-between-each-subject-in-case-of-repeated-measurements

*Figure 5:*

- As with Figure 2 – can you add the test statistic and regression or correlation line to this graph? Also, please add the statistic to Figure 7.

Is the study design appropriate and does the work have academic merit?

Yes

Is the work clearly and accurately presented and does it cite the current literature?

Yes

If applicable, is the statistical analysis and its interpretation appropriate?

I cannot comment. A qualified statistician is required.

Are all the source data underlying the results available to ensure full reproducibility?

Yes

Are the conclusions drawn adequately supported by the results?

Yes

Are sufficient details of methods and analysis provided to allow replication by others?

Yes

Reviewer Expertise:

Host-pathogen interactions, virology, virus ecology, virus evolution, eco-immunology

I confirm that I have read this submission and believe that I have an appropriate level of expertise to confirm that it is of an acceptable scientific standard, however I have significant reservations, as outlined above.

# Response

**Published:** 2021-09-10

**Authors:** Dieter Heylen

**Affiliations:** Hasselt University, Belgium

Dear editors and reviewer,  on behalf of all authors, I am glad to re-submit the manuscript ‘Ineffective humoral anti-tick IgY-response in birds: reaction against pathogen constituents?’. Songbirds are central elements in the ecological networks of ticks, but are heavily overlooked when it comes to elementary biological mechanisms like immune responses against ticks and tick-borne diseases. No studies so far have related individual variation in songbird adaptive immune responses to components of ectoparasite fitness. Our study therefore can be considered as a primer for future work exploring tick epitopes that can be targeted by bird immune components. In the following sections, we state point-by-point how we have dealt with the comments of the reviewer. Adjustments can be traced by ‘track changes’ formatting and alterations.

**Comments to the reviewer**

*The authors aimed to understand the immune response of Blue tits to tick infestation using an experimental approach. Overall, this is an interesting paper, but currently some shortcomings in data presentation (both text and writing) make it hard to understand the details and interpret of the findings.*

*I think a bit more careful explanation is needed for the reader to understand the idea of “tick resistance”. Specifically, that it's not the prevention of ticks infesting birds, but rather a decrease in the impact of ticks on birds (eg, lower tick engorgements and perhaps a more effective immune response).*

 *** We agree with the reviewer we should emphasize that we investigated the processes after the ticks have been placed on the bird’s skin. Furthermore, we realize we could have done a better job in introducing the general idea of the study and its design. Therefore, we added more information in the first sentences of the Introduction’s last paragraph, where the purpose of the study has been made clear, which reads as follows: “This study focuses on the avian adaptive humoral immune response against ixodid ticks as a potential driver of the among-bird variation in feeding success. Hereto, we follow-up ticks that have been placed on the birds’ skin, giving them the maximum opportunity to feed. As the development of an effective immunological response needs time, we exposed birds three times in succession over a time period of 25 days.  Throughout the experiment the birds’ physiological health and IgY-antibody response were monitored (see ‘Methods’ section)”.

 *** In addition, we screened the text for ‘resistance’ and found that it has been used once without ‘anti-’, and a couple of times where it would have been better to place ‘immunological’ in front of it.

*Second, humoral immunity develops following the acute response (inflammation). How do these findings link into studies assessing inflammation following tick infestation? Would this variation in IgY perhaps be due to a variation in acute responses? I recognise that the authors did not measure this, but I wonder if there is data from previous studies?*

 *** The reviewer put forward an interesting hypothetical explanation that may explain the individual variation among birds in IgY responses. We do not have the required data to test this hypothesis, in that the more reactive birds (in terms of inflammatory response) also elicited the highest IgY responses. We mention that inflammatory reactions by the host can be expected after rupture of the integument (the skin) and exposure to novel epitopes.  Remarkably, ticks are one of those ectoparasites that are specialized in maximally reducing those inflammatory responses, making the interpretation of inflammatory reactions a difficult task.  Ticks evade the host repertoire of immunopharmacological agonists by counter-producing a number of salivary antagonists (Ribeiro, 1989 ). We also want to emphasize that ELISA’s are designed to detect antibodies that specifically bind to tick-antigens.  Given the birds have never been exposed to ticks before, it is expected that proteins belonging to the inflammatory reaction/acute response are driving the observed IgY-patterns. Most of the elements given above are already in the manuscript, but we added a line in the ‘Discussion’ section: “the observed IgY-response gives evidence for an adaptive response, rather than an acute inflammatory reaction.”

*Third, as these were field collected ticks, how do the authors control for things like viral infections which may affect Hct or body condition. These viral infections may also affect Engorgement weights and feeding durations of ticks (particularly for viruses infecting the ticks, not necessarily arboviruses which are transmitted to the birds). Theoretically they shouldn’t affect the IgY as this is specific. I recognise that the authors did not measure this, but it is worth point out in the discussion.*

 *** This is an important point, indeed. Therefore, in the last paragraph of the discussion, we added the following sentence “Are anti-tick immune responses, host physiology and tick feeding performance affected by the pathogens themselves and/or other tick associated micro-organisms (including viruses)? Are these responses comparable in different tick-pathogen-host systems? …  All these questions are heavily unexplored in birds.”

    

**Specific comments:**

**Results**

*“Sera I. Ricinus-exposed birds and…”:* *I would encourage the authors to open the first sentence with the purpose of this experiment. For example, “In order to address XXXX, we three tits were monitored at six time points (as opposed to 3). In these birds we found….” Similarly for the next paragraph. “Following this, 10 blue tits (including or excluding the 3 before?) were monitored at three time points. These time points corresponded to direction prior to infestation, and thus reflecting the IgY levels following the previous infestation event. In these birds, OD profiles were….”*

 *** We now have re-organized this entire section.

*“IgY correlations with anti tick resistance”:* *The opening of the second paragraph could improve. Something like: “In order to investigate whether XX influenced YY, we utilised a GLMM. Neither age etc...”  Also, please help the reader a bit more. I’m not sure why “Inf 1” is cross-sectional and “delta Inf3-Inf 1" is longitudinal. Can you explain here what these values mean (especially given Figure 1 is not at all intuitive).*

 *** The problem with the interpretation of Figure 1 should be alleviated, given the improvements made. (1) Figure 1 provides an extensive explanation of the all response variables. (2) Inf (infestation) and D(ay) are now more often written in full (3) with symbols we refer in the explanatory section to the design figure (response variables). In addition, these symbols are used in 2 other figures (Figure 3 and Figure 6) for a better understanding.  

*So, based on this analysis, what did influence IgY levels? Perhaps add, “In sum, none of the factors we tested influenced IgY levels across either each infestation, or across all three infections”.*

 *** We added the suggested sentence.  

*IgY correlations with tick virulence”:* *This section is not well written – its rather written like a list of bullet points rather than a “story” whereby the reader is walked through the analysis with care. For example, adding leader statements like “In order the assess the acute effects, i.e., the variation in Hct levels, XXX YY” are useful.*

  *** We have rewritten the paragraphs and agree that without repeating important elements described in the ‘Methods’ section, this part of the ‘Results’ section is tough to follow.  

*Why were these data points deemed outliers – was this a technical problem?*

  *** Normality assumptions were violated. We mention that both outliers belonged to the same bird individuals.  

*Why were the IgY levels summed?*

  *** The cumulative IgY response has been defined as a proxy for the total investment in specific IgY-antibodies by the bird throughout the course of the experiment.  

*RE Body condition: Perhaps rephrase to say something like “Body condition...for all birds, with the exception of infestation sessions wherein we found an acute effect (here explain which sessions those were).” I also don’t understand why there is reference to chronic effect here?*

 *** We integrated the section on body condition with the section in which the model on Hct is described.  We thus removed the section the reviewer is referring to.  We also removed the reference to chronic.  

*Paragraph 4: Does this mean that you did the analysis twice – once including and once excluding the Birds in Figure 3 (i.e., the birds that were measured 6 times and therefore did not have enough sera remaining to be on all plates)?*

 *** Yes, that is correct.  We removed the sentence, to make the paragraph less complicated. The IgY measurements of the 3 birds were well situated within the measurements of the other 10 birds, and the birds have been treated in exactly the same way throughout the experiment (the reason why putting them together with the other 10 birds in a single analysis makes sense).    

*Paragraph 5: Again, more common language interpretations would be useful here. Why did you use summed values? Why does Hct and Body condition refer to chronic? See comments above about including a leading sentence.*

*** We rewrote the sentences for ‘Chronic effects’.

 *** Now, better explained in Figure 1: “Chronic effects: The health effects of 24 ticks feeding over a time period of 21 days. It is calculated as the difference between the measurements at the moment when birds were still tick-free in Inf. 3 (Day 21) and those at the start of the experiment (i.e. the baseline measurements before the first tick exposure, Day 1).” 

**Discussion**

*Paragraph 2 RE maternal antibodies: Are there studies you can reference that have quantified how long maternal antibodies persist in birds. For example, this has been done in ducks for antibodies against influenza A, but I wonder if this has been done for any passerines for any virus/bacteria? This data is useful for future studies such that you may start experiments when birds are older, and you are sure that the effect of maternal antibodies has waned **.***

 *** After delving deeper into the literature, we found an important study by King et al (2010) entitled “Are Maternal Antibodies Really That Important? Patterns in the Immunologic Development of Altricial Passerine House Sparrows (Passer domesticus)”, stating that  “Based on the short half-life of maternal antibodies, the rapid production of endogenous antibodies by nestlings and the relatively low transfer of maternal antibodies to nestlings, our findings suggest that altricial developing sparrows achieve immunologic independence much earlier than precocial birds”.  The authors estimated the half-time of maternal anti-bodies in nestling plasma as 2.2 +/-0.25 days; they also estimated the immunologic independence as 8–10 days after hatch.  If the blue tit – also an altricial passerine – shows similar physiological patterns, then the IgY levels observed in our study should have been synthesized by the bird itself, as all maternal antibodies should have been cleared from the plasma. We added some extra sentences in the first paragraph of the ‘Discussion’ section, referring to this study: “However, the immunological processes in the early development of altricial birds (among which blue and great tits) differ from those of semi-precocial birds (to which gulls belong). In an experimental study by King et al. (2010) where House sparrows (Passer domesticus L.) are experimentally exposed to novel antigens, the authors observed short half-life of maternal antibodies (less than 3 days after hatching), low transfer from mother to nestlings, and a rapid production of endogenous antibodies by nestlings (8-10 days after hatching).  They concluded that altricial developing birds achieve immunologic independence much earlier than precocial birds, implying that the variation in (initial) IgY levels observed in our study is synthesized by the juvenile bird itself.”  

*Paragraph 3 RE tick resistance: Can the authors please provide an example of hosts types that have been shown to acquire resistance to ticks?*

  *** Typically, development of resistance occurs in artificial tick-host interactions, i.e. that are never observed in nature. We added: “Acquired resistance against ticks has frequently been demonstrated in laboratory rather than in natural hosts, therefore it has been suggested that tick resistance is confined to artificial host–tick associations (e.g.  guinea pigs infested with *Dermacentor variabilis*, rabbits infested with *I. ricinus*) (Bowessidjaou et al., 1977; Ribeiro, 1989; Wikel 1996) and that successful parasitism in natural host-tick associations is the result of an intense co-evolution, in which ticks developed adaptations to evade the host’s immune system (Ribeiro, 1989; Fielden et al., 1992)”.  

*Also, you have a statement: “We conclude that naïve juveniles do not acquire anti-tick resistance”. Do adult passerines acquire this resistance? Are you sure you are using the correct measurements and experimental design for this as it is a strong statement to make?*

 *** We are sure about the use of the correct measurements: tick engorgement weight is considered to be one of the most consistent indicators of the host’s immunological anti-tick resistance. More than likely adults do not acquire immunological anti-tick resistance: the many experiments we have executed in our lab with a diverse set of natural host x tick interactions, all resulted in high feeding success, especially with regard to engorgement weights (great and blue tits, as well as blackbird, infested with *I. ricinus*, *I. arboricola* and *I.frontalis*). We mention that most of these experiments took place in different seasons (autumn, winter, early spring) than the current study.

 *** We added ‘immunonlogical’ to the sentence, which reads as follows “We conclude that naïve juveniles do not acquire immunological anti-tick resistance.”. We have some indications that adult passerines – given their past experience with ectoparasite exposures – became better in grooming than young birds, lowering the proportion of successfully attached ticks (not the engorgement weights).  

*Paragraph 3 RE tolerance. Given fitness wasn’t really measured and the experimental design limited and controlled for tick infestations, I am not sure how convincing this is. Are there are examples that the authors could reference for animals that tolerate ticks?*

 *** The concept of tolerance is notoriously difficult to measure in animals. As shown by Raberg and colleagues (cited in the paper), the demonstration of tolerance requires experimentally infecting replicates of different host genotypes with different loads of pathogens and measuring host fitness. Tolerance for each host genotype is then estimated as the slope of how host fitness decreases with pathogen burden. Host genotypes that have a flat slope between host fitness and pathogen load are considered tolerant. All this, is very hard to measure in a single experiment (and to the best of our knowledge, this has never been tested in ticks x vertebrate systems). However, controlled experiments have shown that adult birds with and without tick infestations – with tick burdens close to the upper limit observed in nature – did not differ in breeding success (Heylen et al. 2009). Moreover, birds infested in captivity showed no decrease in body condition compared to control birds, despite the measurable drop in haematocrit. We also note that in the experiment reported here, tick burden did not affect mortality (Heylen et al. 2010). Although additional experiments with varying tick burdens and including long-term survival would be required to fully support the concept of tolerance as it was defined by Raberg and colleagues, we want to leave the following sentence in the manuscript: “Here, high burdens did not cause direct fitness effects (bird mortality) or gave rise to indirect fitness effects via physiological measure that link up with bird fitness; both findings are in favour of tolerance”. But in order to tone down our previous (hypothetical) conclusion, we added a sentence in the last paragraph of the ‘Discussion’ section: “Does also the bird’s long-term fitness remain unaffected by high tick loads, providing further indirect evidence for tolerance (as defined by Råberg and colleagues (2009))?”

**My suggestions for Figure improvement:**

*Figure 1:* *Concept figures should be immediately understandable without needing to read the legend, but unfortunately Figure 1 is very confusing. There is plenty of space so I suggestion you write out “Inf. 1” and “D1”. The legend isn’t very clear either. The figure doesn’t really explain well the different between “Acute” and “Chronic”. Overall, I suggest reconsidering this figure – there are lots of great concept figures out there in papers that rely on infection experiments for inspiration.*

 *** We have the opinion that without text, it will be very hard to understand the different contrasts (acute, chronic, change in acute effects) that were calculated in the different statistical models.  Instead of re-drawing the time-line of the experiment, we now more extensively explained how the contrasts have been defined.   

*Figure 2: Based on the Figure and the legend, it is unclear to me the purpose of this figure. Perhaps a more descriptive title such as: Correlation between first and second IgY measurement of the same sample/bird”. Can you please add the Statistic onto the figure, and also the line. I expect that you could leverage more colours to make it easier for the reader.*

 *** The purpose of the figure is to show that our assay is characterized by a relatively high repeatability. As far as we know, outcomes from this assay - with salivary gland extracts and songbird serum - have not been published before, the reason why we wanted to have the figure in the manuscript.  The correlation coefficient, as a measure of repeatability, is given in the text and now in the figure as well.  Symbols and shapes identify each of the groups: control birds, and experimentally infested (both in which several bird species were included). These have been described in detail in the ‘Methods’ section.

 *** We prefer not to add P-values and test statistics in the figures, as several of the statistical analyses were far more advanced than a least-squares regression.  Instead, to address the comment of the reviewer, we added a Pearson-correlation coefficient: a descriptive measure of association.  

*Figure 3: Again, I would change the figure legend so that this plot is easier to appreciate. For example: Change in IgY levels of 3 Blue Tits that were sampled on 6 occasions. Given Figure 1 isn't intuitive, I still don’t really understand this plot. Perhaps add some extra metadata below the bars – lines or square brackets to indicate infestation periods, or different colours or arrows? Were the positive and negatives only for Day 1?*

 *** We extended the legend with “Positive and negative control birds: repeatedly exposed great tit and naïve canary, respectively.”, making clear that those additional samples have been included, needed to show the response in completely naïve birds (among which the Canary finches which have never been in the wild, thus never been exposed to ticks) and heavily infested birds (great tits, see ‘Methods’ section).        

*Figure 4.: I would suggest that this plot be a boxplot or point graph, with day on the x-axis, and the different birds in different colours.*

 *** A boxplot has been generated, including a line that connects the means of the IgY-measurements based on the ELISA’s with *I. ricinus* antigens. The sera used for cross-reaction (read: IgY-measurements based on the ELISA’s with *I. arboricola* antigens) has been placed in its chronological position, though not connected with the other box plots, given it depicts a different reaction from the others. *** We mention that in the ‘Discussion’ section (as well as in the ‘Results’ section), we referred to initial values of certain bird individuals for which the IgY levels were relatively high.  In order to provide the reader with a graphical overview of the raw data, we kept the previous Figure 4, but placed it in the ‘Supplementary materials.’   

*Figure 5: As with Figure 2 – can you add the test statistic and regression or correlation line to this graph? Also, please add the statistic to Figure 7.*

 *** Correlation coefficients have now been added, as well as a least squares regression line.

        

 On behalf of all authors,  Sincerely Yours,   Dieter Heylen
